# NprR, a moonlighting quorum sensor shifting from a phosphatase activity to a transcriptional activator

**DOI:** 10.15698/mic2016.11.542

**Published:** 2016-11-04

**Authors:** Stéphane Perchat, Antoine Talagas, Samira Zouhir, Sandrine Poncet, Laurent Bouillaut, Sylvie Nessler, Didier Lereclus

**Affiliations:** 1 Micalis Institute, INRA, AgroParisTech, Université Paris-Saclay, 78350 Jouy-en-Josas, France.; 2 Institute of Integrative Biology of the Cell (I2BC), CEA, CNRS, Univ. Paris-Sud, Université Paris-Saclay, 91198 Gif-sur-Yvette cedex France.

**Keywords:** Bacillus, bifunctional protein, phosphatase, quorum sensing, sporulation

## Abstract

Regulation of biological functions requires factors (proteins, peptides or chemicals) able to sense and translate environmental conditions or any circumstances in order to modulate the transcription of a gene, the stability of a transcript or the activity of a protein. Quorum sensing is a regulation mechanism connecting cell density to the physiological state of a single cell. In bacteria, quorum sensing coordinates virulence, cell fate and commitment to sporulation and other adaptation properties. The critical role of such regulatory systems was demonstrated in pathogenicity and adaptation of bacteria from the *Bacillus cereus* group (i.e. *B. cereus* and *Bacillus thuringiensis*). Furthermore, using insects as a model of infection, it was shown that sequential activation of several quorum sensing systems allowed bacteria to switch from a virulence state to a necrotrophic lifestyle, allowing their survival in the host cadaver, and ultimately to the commitment into sporulation. The chronological development of these physiological states is directed by quorum sensors forming the RNPP family. Among them, NprR combines two distinct functions connecting sporulation to necrotrophism in *B. thuringiensis*. In the absence of its cognate signaling peptide (NprX), NprR negatively controls sporulation by acting as a phosphatase. In the presence of NprX, it acts as a transcription factor regulating a set of genes involved in the survival of the bacteria in the insect cadaver.

In Gram-positive sporulating Bacilli, triggering of the sporulation process requires a certain threshold concentration of the transcriptional regulator Spo0A-P, whose phosphorylation depends on the activity of a multicomponent phosphorelay controlled by Rap phosphatases. The Rap proteins are quorum sensors belonging to the RNPP family and their activity is inhibited by the Phr signaling peptides. The Rap proteins involved in the sporulation phosphorelay dephosphorylate the component Spo0F-P. The interruption of the phosphorylation cascade reduces the concentration of Spo0A-P in the bacterial cell and impedes sporulation. This negative effect is relieved when the Phr peptide binds to the Rap protein.

Like other RNPP quorum sensors, the peptide-binding domain of the Rap proteins is characterized by the presence of repeated motifs consisting of a 34 amino acid degenerated sequence folded into two antiparallel α-helices and called tetratricopeptide repeats (TPRs) involved in protein-protein interactions. In addition, with the exception of Rap proteins, RNPP regulators contain an N-terminal helix-turn-helix (HTH)-type DNA-binding domain and act as transcriptional regulators. By contrast, Rap phosphatases interact with their target proteins via two additional N-terminal TPR motifs. Interestingly, the regulator NprR contains both the HTH DNA-binding domain and the Rap-like additional TPR motifs. Among all the transcriptional regulators of the RNPP family, sequence comparison clearly shows that NprR is closely related to the Rap proteins, and can thus be proposed as an evolutionary intermediate in the RNPP family (Fig. 1).

**Figure 1:**
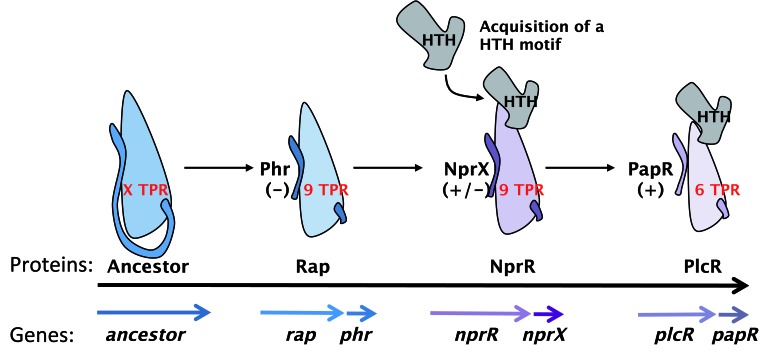
FIGURE 1: Evolution of the RNPP family in Bacilli. The presumed ancestor protein contains X TPR motifs and an inhibitor C-terminal end. The first step of this evolution scheme is the separation of the ancestral gene in two separate genes encoding, from 5’ to 3’, the quorum sensor (Rap) and an inhibitor signaling peptide (Phr). The second step is the acquisition of an HTH domain resulting in a bifunctional protein (NprR) controlled by a molecular switch (NprX) having an inhibitory (-) or activating (+) effect. The third step is the loss of 3 TPR motifs and leads to the PlcR-PapR quorum sensing system.

The phylogenetic similarity between NprR and Rap was confirmed by a structure-function analysis. We have shown that NprR binds and dephosphorylates Spo0F-P like a Rap phosphatase, thus inhibiting the phosphorylation cascade involved in the activation of Spo0A. This effect abolishes the expression of Spo0A-regulated genes and significantly reduces the sporulation efficiency of the bacterial population. In a *B. thuringiensis* mutant strain unable to produce NprX, the production of heat-resistant spores is reduced about 2000-fold compared to the *B. thuringiensis* wild-type strain. Altogether our results clearly demonstrate that the apo form of NprR is a Rap-like phosphatase. Moreover, we have shown that this activity is relieved by NprX binding: the NprR-NprX complex binds DNA and activates the transcription of 41 genes forming the NprR regulon. This transcriptional activity of NprR allows the bacteria to enter into a necrotrophic lifestyle and thus to survive into the cadaver of the invertebrate host.

From the structural point of view, we showed that the apo form of NprR is a dimer displaying a highly flexible Rap-like structure. A mutational analysis based on sequence comparison suggests that the Spo0F binding mode is conserved with the Rap proteins. The N-terminal extension oscillates between an extended 3-helix bundle conformation stabilized by Spo0F binding and a compact TPR conformation stabilized by peptide binding. The resulting NprR-NprX complex associates into a tetramer forming two pairs of HTH domains that most probably bind two DNA target sequences in a cooperative manner, as suggested for PrgX, another tetrameric RNPP regulator.

A study of the cell fate in a *B. thuringiensis* population infecting an insect larva demonstrated that commitment to sporulation arises only from bacteria engaged in necrotrophism, i.e. from bacteria in which NprR acts as a transcriptional activator. This result indicates that the Rap-like function of NprR should be switched off to allow the bacteria to reach the threshold of Spo0A-P required for triggering the sporulation process. The NprR-NprX system establishes a very strict coupling of two physiological stages, necrotrophism and sporulation; thus preventing sporulation of bacteria that are not formerly engaged in a necrotrophic lifestyle. In other terms this coupling suggests that the bacteria should enter into necrotrophism to take a maximal advantage of the nutriments available in the host cadaver before irreversibly committing to sporulation. This strategy may ensure the most efficient survival and dissemination of the bacteria during the infection process. Moreover, the use of a bifunctional protein to combine and direct necrotrophism and sporulation prevents any defect of synchrony and any inconsistency between these two essential physiological stages. The moonlighting NprR protein is a good illustration of the French proverb: you are never better served than by yourself.

